# Accelerating Formulation Design via Machine Learning: Generating a High-throughput Shampoo Formulations Dataset

**DOI:** 10.1038/s41597-024-03573-w

**Published:** 2024-07-03

**Authors:** Aniket Chitre, Robert C. M. Querimit, Simon D. Rihm, Dogancan Karan, Benchuan Zhu, Ke Wang, Long Wang, Kedar Hippalgaonkar, Alexei A. Lapkin

**Affiliations:** 1 Department of Chemical Engineering and Biotechnology, University of Cambridge, Philippa Fawcett Drive, Cambridge, CB3 0AS UK; 2 Cambridge Centre for Advanced Research and Education in Singapore, CARES Ltd. 1 CREATE Way, CREATE Tower #05-05, Singapore, 138602 Singapore; 3 Institute of Materials Research and Engineering, Agency for Science, Technology and Research (A*STAR), Singapore, 138634 Singapore; 4 School of Chemistry, Chemical Engineering and Biotechnology, Nanyang Technological University, Singapore, 637459 Singapore; 5 BASF Advanced Chemicals Co. Ltd., No. 300, Jiang Xin Sha Road, Pudong, Shanghai, 200137 China; 6 School of Materials Science and Engineering, Nanyang Technological University, Singapore, 639798 Singapore

**Keywords:** Fluids, Physical chemistry

## Abstract

Liquid formulations are ubiquitous yet have lengthy product development cycles owing to the complex physical interactions between ingredients making it difficult to tune formulations to customer-defined property targets. Interpolative ML models can accelerate liquid formulations design but are typically trained on limited sets of ingredients and without any structural information, which limits their out-of-training predictive capacity. To address this challenge, we selected eighteen formulation ingredients covering a diverse chemical space to prepare an open experimental dataset for training ML models for rinse-off formulations development. The resulting design space has an over 50-fold increase in dimensionality compared to our previous work. Here, we present a dataset of 812 formulations, including 294 stable samples, which cover the entire design space, with phase stability, turbidity, and high-fidelity rheology measurements generated on our semi-automated, ML-driven liquid formulations workflow. Our dataset has the unique attribute of sample-specific uncertainty measurements to train predictive surrogate models.

## Background & Summary

Liquid formulations are ubiquitous in everyday lives and are produced across several industries, such as cosmetics, food, pharmaceuticals, and agrochemicals^1,2^. Increasingly, there is demand for a switch to eco-friendly products^3^. For example, there is a push to replace polymers in liquid formulations (PLFs), which are often made from fossil-derived monomers, with natural polymers, or those derived from bio-feedstocks^4,5^. Since designing liquid-formulated products that meet multiple customer-defined property targets is a time-, resource- and labour-intensive process^6^, changing the carefully optimised formulation recipes is a difficult proposition. In this respect, developing accurate predictive models, either through computational approaches like machine learning (ML)^7–11^ or molecular modelling simulations^12–14^, can aid in accelerating formulation design.

In an earlier study, some of us have developed interpolative ML models, trained on a relatively small dataset of liquid formulations^7^. This earlier study showed the potential of ML models to learn complex interactions within a formulation and the use-case within a workflow of formulations design. However, the models, trained on a limited set of formulation ingredients and without structural information about said ingredients in the input features, are rather limited in scope and cannot be generalised to new formulations without re-training the model on a new experimental dataset. For developing statistical-ML models, availability of experimental data to train or validate the models is the most critical factor^3,15,16^.

In this study, we aim to create an open dataset for training machine learning models in the liquid formulations domain. Our chosen liquid formulation system is shampoo, similar to the previous study by Cao *et al*.^7^ We selected twelve surfactants covering anionic, non-ionic, amphoteric, and cationic types^17^, four conditioning polymers, and two thickeners. From these we chose a binary mixture of surfactants, polymer, and thickener in any given formulation. These ingredients are also more broadly used in rinse-off product applications. Due to the combinatorial nature of formulation design, our set of ingredients lead to 528 distinct combinations, an over 50-fold increase in dimensionality compared to our previous study^8^. Further to the choice of ingredients, we investigated a diverse range of ingredient concentrations.

With a large range of ingredient choices and concentrations to explore and a limited experimental budget, an efficient and cost-effective high-throughput (HT) workflow needed to be developed. Such workflows are becoming increasingly important across a wide spectrum of domains, including, but not limited to, organic chemistry^18,19^, inorganic materials^20,21^, and biological applications^22^. The convergence of ML, lab automation and robotics has led to the term “self-driving labs” (SDLs) being coined^23^.

Automation brings along several benefits: increased efficiency, reproducibility, and safety; but not everything can be integrated into an automated workflow, in particular, certain analysis techniques represent the bottleneck^24^. As an alternative, if a particular characterisation instrument cannot be integrated with the synthesis set-up, samples can be brought to the analytical instruments, as in the case of the *mobile chemist robot* developed in the Materials Innovation Factory (MIF) at the University of Liverpool^25^. However, there is a very significant capital investment, both monetary and human, required to setting up such a laboratory. Instead, this work favours the approach of flexible, modular automation by breaking down the process of preparing and characterising formulation samples into unit operations which we aimed to automate with independent stations for liquid handling, pH adjustment, analysis *etc*^21,23,26^.

There are a couple of interesting commercial formulation bots such as GEOFF by Labman Automation^27^ (located at the University of Liverpool, with industrial backing from Unilever) and FORMAX by ChemSpeed Technologies^28^. One option we had was to buy time on an already available bespoke robotic platform, for example within the MIF at Liverpool. The alternative was to develop a workflow that demonstrates how any organisation with a not-too-large, dedicated lab automation budget, could make use of their existing stand-alone systems and complement them with lower-cost robotics/automation.

**Fig. 1:**
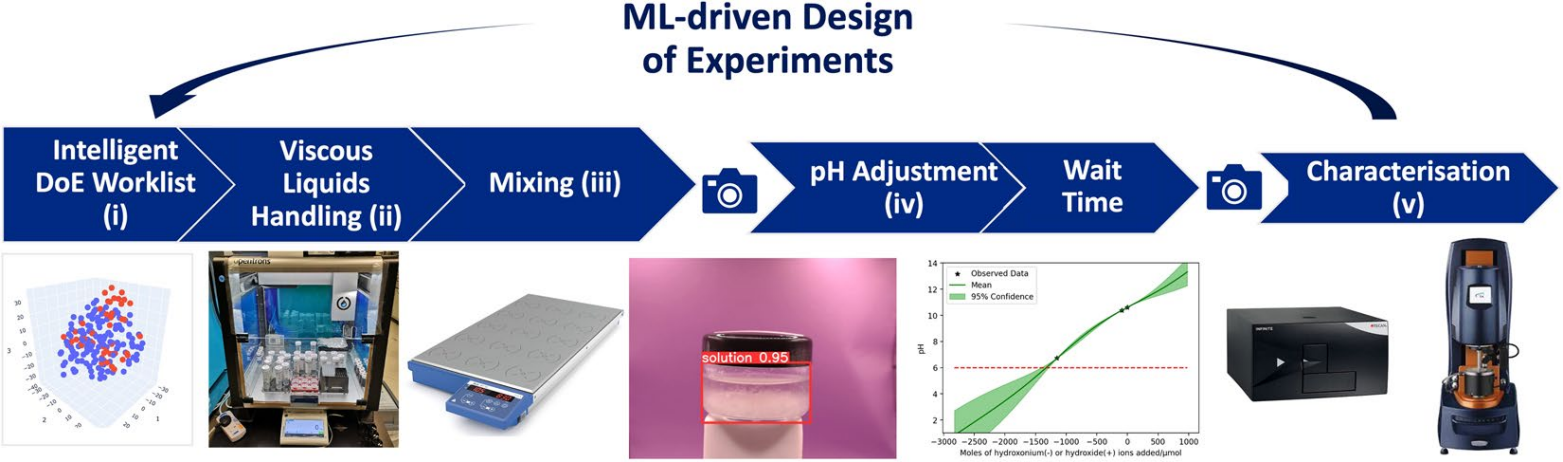
An overview of the liquid formulation workflow. (i) From an ML-guided design of experiments^29^; (ii) preparation of the liquid formulations by automated viscous liquids handling^30^; (iii) mixing on a multi-position stirrer plate; followed by (iv) pH adjustment on the pHbot^31^; and finally, (v) characterisation including optical imaging for phase stability, proxy UV-vis absorbance for turbidity, and rotational rheometry for viscosity measurements.

Figure 1 shows our semi-automated liquid formulations workflow, where we adopted a “human-in-the-loop” approach to transfer samples between workstations, with only some steps being automated unit operations. The first step of the workflow was a ML-guided design of experiments (DoE), which is outside the scope of this Data Descriptor and is detailed elsewhere^29^. The DoE output was read by an Opentrons OT-2 liquid handling robot which we retrofitted with a mass balance for automated viscous liquid handling^30^. After this step, the formulations were mixed and characterised for their phase stability. We carried forward the stable samples to our pHbot^31^ for pH adjustment, and once again, after a 36-hour wait (an agreed processing step), assessed stability of our products, before we measured turbidity and viscosity of the stable formulations. Rheology measurements were performed offline, as they are very difficult to automate, particularly for highly-viscous and non-Newtonian fluids^32^.

We generated over 800 formulations within eight months using this workflow and instrumentation. We made the choice of samples to be chemically diverse, exploring different chemical functionalities of typical ingredients and their concentration ranges, to maximise the utility of the resulting dataset for training ML models for formulations properties prediction.

The remainder of the paper is organised as follows. We firstly detail the experimental workflow to prepare and characterise the formulations in the Methods section. We then present the formulations dataset as a JSON file for a compact representation in the Data Records, along with a discussion of supplementary files (chemical structures, phase stability images) shared on the dataset repository. Under Technical Validation, we demonstrate the statistical diversity of the dataset and discuss the errors and confidence in our characterisation techniques. Furthermore, we show the utility of the image dataset for training computer vision models. Finally, under Usage Notes, we briefly discuss how to work with the JSON file in a Python Pandas dataframe, which is the most popular format for data science and machine learning.

## Methods

### Viscous liquids handling with a retrofitted opentrons robot

**Fig. 2:**
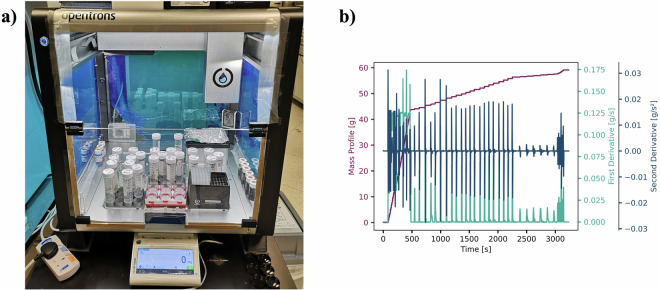
Details of liquid handling of formulations. (**a**) Mass balance integrated with an Opentrons OT-2 liquid handling robot^30^ – pipettes formulation ingredients according to an input CSV file with desired ingredient mass fraction and outputs a CSV file with a mass *vs*. time profile; (**b**) mass profile signal (purple) as well as its first (green) and second derivatives (blue) used by our signal processing algorithm to resolve the formulation compositions.

We prepared our samples from commercial liquid formulation ingredients, which were used as received and are presented under the Data Records. These ingredients ranged from low (~10 mPa·s) to very high viscosities (10,000 mPa·s+) as shown in Supplementary Figure S1. We selected the Opentrons OT-2 liquid handling robot for ease-of-use and customisability of its operating parameters, which is essential for handling viscous fluids. Despite our attempts at optimising the OT-2’s parameters to handle our ingredients^33^, we could not get the liquid handler to dispense the target amounts specified from the DoE. It remains an open challenge to achieve accurate transfers of highly viscous, particularly non-Newtonian, fluids on a pipetting robot. Some of our group recently performed a study into closed-loop optimisation of liquid handling parameters for viscous handling, however, this work only goes up to 1275 mPa·s^34^. Instead, we found roughly optimal liquid handling parameters for each class of ingredient (surfactant/polymer/thickener) and retrofitted the OT-2 with a precision balance, as shown in Fig. 2a^30^. Within this study, we have generated a dataset aimed at training ML models for formulation design. Therefore, instead of needing to be able to pipette our ingredients precisely and accurately, we only needed to accurately *measure* the mass of each ingredient added and could then back-calculate our prepared formulations compositions. The purple curve in Fig. 2b shows a typical mass *vs*. time profile from preparing a batch of six formulations in one Opentrons run. This signal was deconvoluted, using an analysis of the first and second derivatives of the mass profile, to identify the ingredient additions and calculate the formulation compositions. We discuss further details of this method in the Supplementary Information (SI).

### Mixing, imaging, and titrating formulations

We mixed the formulations for 35 minutes at 360 rpm and 25 °C using Fisherbrand™ rare earth octagonal stir bars (25 × 8 mm) on an IKA RT 15 multi-position stirrer plate. The formulations were left to rest for 15 minutes before we visually inspected their stability. Unstable formulations were discarded at this stage, as preliminary tests showed a very low likelihood of an unstable formulation stabilising after pH adjustment. Additionally, titrating unstable formulations is particularly challenging and time-consuming.

We defined a stable formulation as a single homogenous phase with no discernible phase separation, flocculation *etc*. We imaged each formulation using a Logitech C922 webcam controlled by open-cv^35^ (see Supplementary Figure S3) and labelled each image as stable (‘true’) or unstable (‘false’). However, we sometimes could not distinguish a particular sample’s phase stability from the computer image alone and would need to check the sample by eye from different angles, or under different light, and therefore, recorded these images in a separate “borderline” directory (as opposed to “main”). This does not affect the useability of the complete dataset as all the samples have a well distinguished stability label by eye; it is only the supplementary image dataset which is slightly more restricted in size, but still over a thousand images, as “borderline” images have been separated to maintain the image set quality.

Next, we titrated the formulations to pH 5.8 ± 0.2 with citric acid or sodium hydroxide, as per an industrial requirement, using our fully automated pHbot^31^. Our target pH is typical for shampoo formulations^36,37^. After pH adjustment, the samples were left for 36 hours in ambient lab conditions and then re-assessed to record their short-term phase stability. Typically, industry performs longer-term phase stability testing, including at elevated temperatures to accelerate the degradation process. Here, to develop a large dataset to model the formulation’s properties, a short-term test is more suitable to maintain throughput. Only formulations stable post pH-adjustment, *i.e*., during both visual inspections (pre- and post-titration), were recorded as stable in the dataset.

### Formulations characterisation

We carried forward the remaining stable formulations for turbidity and viscosity testing. A Turbiscan would be ideal for high-throughput turbidity analysis and could additionally be used to assess destabilisation mechanisms (*e.g*., creaming, sedimentation, flocculation, and coalescence)^38^. However, we did not have access to such an instrument and nor is it a reasonable expectation, under limited time and budget constraints, to be able to access the ideal choice of equipment at every stage of a workflow. Instead, a degree of creativity is essential to build high-throughput/automated workflows and one trick is to develop fast proxies^39^. Proxy measurements can be employed in both experimental^22,40–42^ and computational work^43^.

We measured UV-vis absorbance at a fixed wavelength (420 nm) as a proxy measurement for turbidity using the Tecan Infinite M Plex 200 Pro plate reader. We used a Corning® 96-well clear flat bottom UV-transparent microtiter plate (MTP). We purchased a range of turbidity standards from Sigma Aldrich (1, 5, 10, 100, 1000, 4000 NTU) and used these, as well as intermediates prepared through their mixtures, to develop a UV absorbance – turbidity calibration curve (Supplementary Figure S4). We transferred 4 aliquots per sample into a MTP so that we had sufficient repeats, as if the formulations foamed during their transfer into the MTP, this would impair the measurement. We applied a local outlier factor (LOF) anomaly detection algorithm (with two nearest neighbour points) and used the average and standard deviation from the remaining points to read off our proxy turbidity measurements and their associated 95% confidence interval from the calibration curve.

Since it is very challenging to affordably automate viscosity measurements^32,41^, particularly for non-Newtonian fluids where we are interested in characterising their viscosity – shear rate profile, we performed high-fidelity rheometry experiments offline. This was the bottleneck of the workflow but let us capture rich data about the formulations. We used two different rheometers with slightly different configurations. While the reason for this was operational, it is, nevertheless, typical of the types of hurdles faced during a large experimental campaign. We used the TA DHR 30 with a 60 mm parallel plate and 250 *μ*m plate gap for S1-618 and S690-822 and the Anton Paar MCR702e with a 25 mm parallel plate and 200 *μ*m plate gap for S619-689. We performed a constant temperature (25 °C) shear-rate sweep between 1–1000 s^−1^. This is representative of pouring to spreading/lathering shear rate range^44^. We performed at least three repeat measurements for each sample and grouped data into two results guided by our domain expertise: i) classifying the hypothetical “zero-shear” viscosity for each formulation as very low (≤10 mPa·s), low (10 < *η* ≤100 mPa·s), medium (100 < *η* ≤1000 mPa·s), or high (>1000 mPa·s); and ii) whether the sample is a Newtonian, shear-thinning, or another type of non-Newtonian fluid. Additionally, we recorded the complete data of shear-rate *vs*. average viscosity measurements, along with their standard deviations.

We include for the knowledge of our community in the SI, an estimated capital cost-breakdown of developing this semi-automated, ML-driven liquid formulations workflow.

## Data Records

The dataset, with 812 liquid formulations, is provided as a JSON file, publicly available at^45^. This cleaned dataset excludes ten samples from the total of 822 prepared formulations (~1% exclusion rate illustrating the high-quality of the workflow) for the reasons stated in Table 1. For the formulations which were too turbid, this would have resulted in extrapolation for the proxy turbidity measurement, which would be unreliable. Additionally, we found it hard to distinguish the stability of these very cloudy formulations. Each data entry in the JSON file corresponds to a sample including information about its ingredient composition and phase stability, plus turbidity and rheology measurements (including the full flow curves) for the stable formulations, of which we prepared 294. Each formulation in the set is a mixture of two surfactants, a polyelectrolyte, and a thickener in a base of water.

**Table 1:** Samples excluded from the cleaned formulations dataset.

Reason for Exclusion	Sample ID
Missing rheology data	26, 209
Too high turbidity (>4000 NTU)	568, 579, 619, 623, 624, 661
Failed pH adjustment (≠5.8 ± 0.2)	750, 787

On the figshare repository^45^, we share the commercial formulation ingredients we used in a slide deck (“BASF Formulation Ingredient Structures”) containing the trade and INCI names for each of the eighteen ingredients, along with their chemical structures. SMILES strings are provided where possible, *i.e*., for all the surfactants and one thickener (Arlypon^®^ F) – the small molecules. The remainder of the molecules are polymers, for which the monomer units, and their proportions (for co-polymers), are included. The estimated molecular weight range is also provided for these macromolecules where available from our industrial partner. Further, to these slides, a separate CSV file (“BASF Surfactants Information”) is provided for the surfactant ingredients, detailing their active matter/water content %, as well as any other content, *e.g*., preservatives (potassium sorbate), or buffering agents (citric acid), which were present for the anionic surfactants. Furthermore, as we worked with real commercial materials, we found via UPLC-MS characterisation (protocol detailed in the SI) that the surfactant ingredients had a distribution of chain lengths, as summarised in Supplementary Figure S5 – the average chain length calculated from these results and a functional group count is included in the surfactants CSV file.

Finally, on the repository^45^, we publish 1,292 formulation phase stability images, including 176 separated into a “borderline” category as discussed above, which we suggest should be excluded from training any ML models, but are included for completeness.

We endeavoured to make this dataset FAIR: i) findable through using a consistent and unique sample ID for each formulation; ii) accessible by sharing the dataset on a public repository; iii) interoperable through the use of a common and system-agnostic data format (JSON), and iv) reusable with an open licence to use this dataset and thereby follow the FAIR community guidelines for data management^46,47^.

## Technical Validation

Within this section we discuss the experimental uncertainties resulting from our methods and illustrate the utility of this dataset. We present our discussion in the order of the workflow.

### Chemical diversity of the dataset

**Fig. 3:**
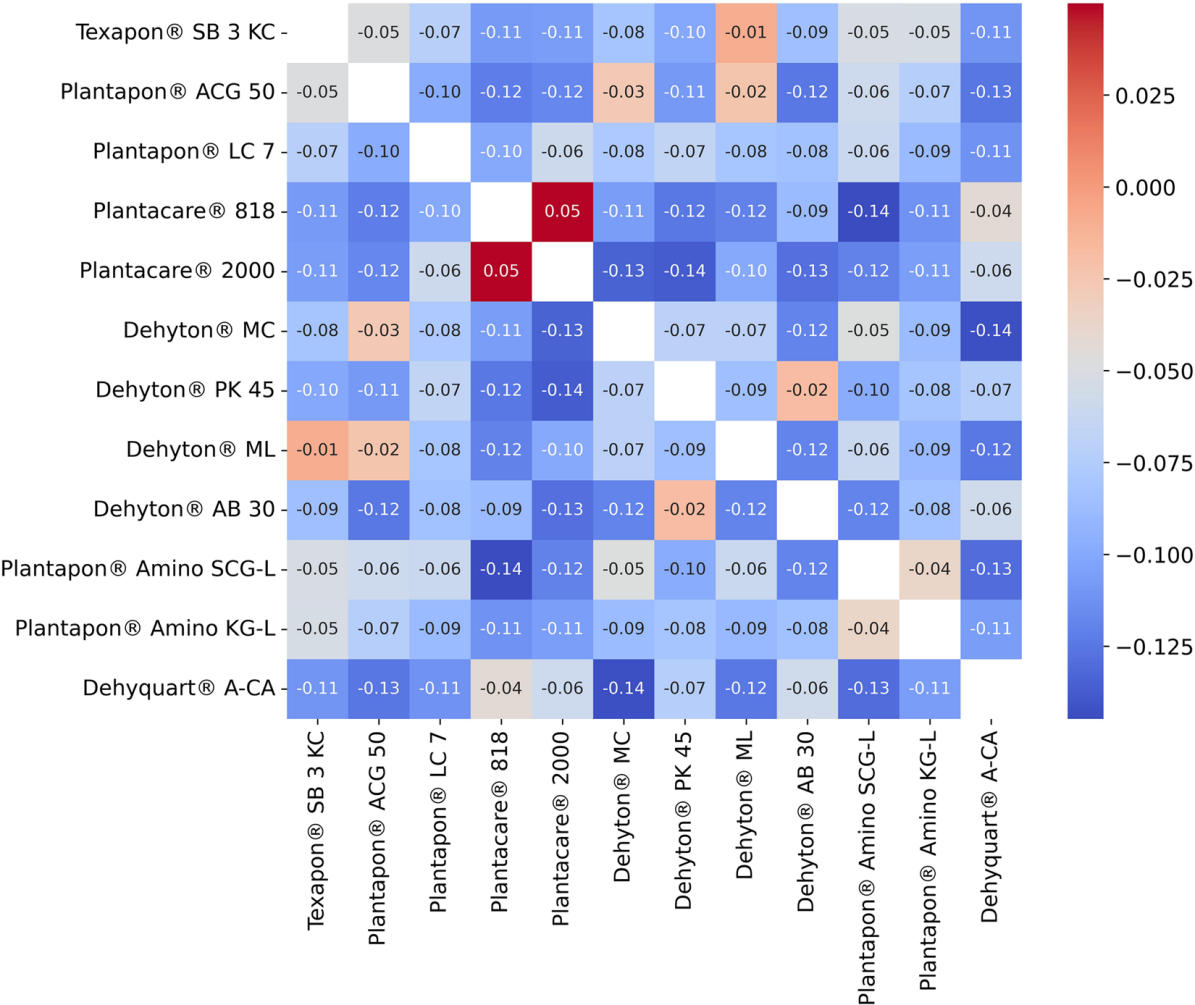
A heatmap for the surfactant ingredient concentrations across the formulations dataset.

Our automated viscous liquid handling method is self-validating by design, as we have incorporated a balance with the OT-2 to measure mass of ingredients added; therefore, we confidently know compositions of the prepared formulations. Figure 3 shows a correlation heatmap as evidence of statistical diversity of the generated dataset.

As mentioned under the Background & Summary, the DoE method used for this work is outside the scope of this article and is properly detailed elsewhere^29^. Briefly, an ML-guided space-filling design was used with the goal to maximise the coverage and spread in the dataset (and explore towards regions of phase stability), so that the complete formulations space is represented. This subsequently allows accurate prediction across the entire design space. There are eight unique combinations of polymer and thickener used in this study and we cycled through each combination, focusing the DoE on one subsystem (polymer, thickener combination) at a time. Figure 3 shows that the surfactant compositions are not correlated with one another, and therefore, that the dataset is not biased to any one region of the design space. This is further supported by Supplementary Figures S6, S7, which show histograms for the surfactant ingredient concentrations and a pair plot of all the formulation ingredients across the dataset. We see a well-distributed and non-correlated spread for the formulation compositions.

### Computer vision for phase stability classification

**Fig. 4:**
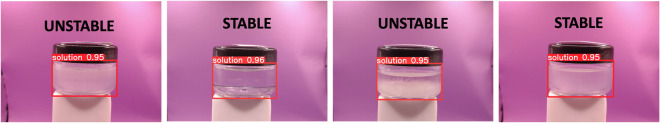
Representative sample images for phase stability classification. The red bounding boxes show the part of the image identified by the YOLOv5s object detection model as the region of interest, the liquid formulation, which is to be classified as stable or not.

To demonstrate the utility of the image dataset, we trained a computer vision (CV) model for phase stability classification. There is an apparently increasing adoption of CV, including in the formulations domain^48–50^. Figure 4 shows a small selection of sample images which the CV model must classify. We developed a two-stage method, with a first stage of identifying the formulation in the image, *i.e*., object detection, and then the second stage performing the classification. We use the popular YOLOv5s^51^ (“you only look once”) object detection model trained on a subset of the first 300 images we generated, from which we can train an almost perfect crop detection for our formulations: 0.995 mean average precision (mAP) at a 0.5 intersection over union (IoU) threshold. We introduce this crop detection prior to the classification, as it improves the final model performance.

We then use a standard 3-layer convolutional neural network architecture (with 16, 32, 64 filters in each convolution layer) to train the phase stability classifier, using the full set of images and only holding out the final sub-system investigated (formulations prepared with polymer = Dehyquart^®^ CC7 Benz and thickener = Arlypon^®^ TT which represent ~10% of the image dataset). The overall test-set performance of this two-stage model is a 0.84 F_1_ score (precision = 0.92, recall = 0.77), which is a reasonably strong classifier, particularly when one considers the ML model has a challenging task of distinguishing some weak phase boundaries through transparent labware. This is an open challenge in the community^52,53^ and we invite computer vision experts to try and improve on our benchmark. We envisage the application of this work in a smart R&D laboratory where a similar webcam could be set up to analyse a conveyor belt of formulations, where the stable ones are carried forward for further processing, as in this study, and the others are safely discarded.

### Turbidity and Rheology measurements

**Fig. 5:**
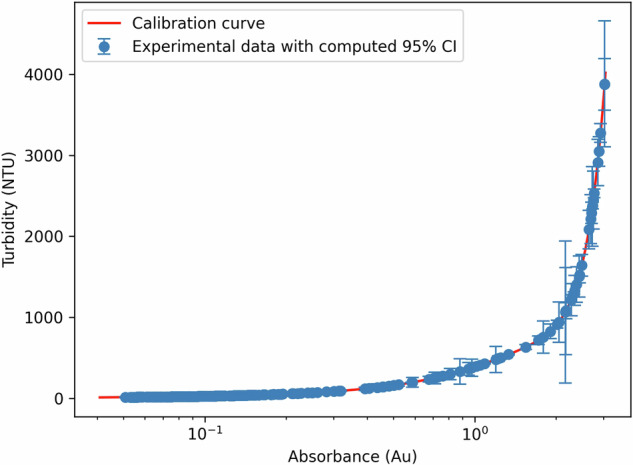
Liquid formulations proxy turbidity measurements.

We now discuss the experimental uncertainty for our two main characterisation measurements in this study, turbidity and viscosity. The turbidity calibration curve, highlighted in red in Fig. 5, with the original calibration points in Supplementary Figure S4, has a R^2^ value of 0.997. Figure 5 shows the liquid formulations’ proxy turbidities computed from their measured UV absorbances at 420 nm, along with their 95% confidence interval. As expected, we see a linear relationship between turbidity and absorbance at low turbidity. However, at higher turbidities, far fewer photons can pass through to the UV-vis detector, so we see a sharp rise in the calibration curve. This is the primary factor for the larger uncertainty at higher turbidity values, as the same absolute uncertainty in absorbance measurement will result in a larger uncertainty in the proxy variable. Except for a few points, there is a low (<10%) uncertainty for most reported measurements, particularly, at the lowest turbidities, which is the region of greatest interest for formulators.

**Fig. 6:**
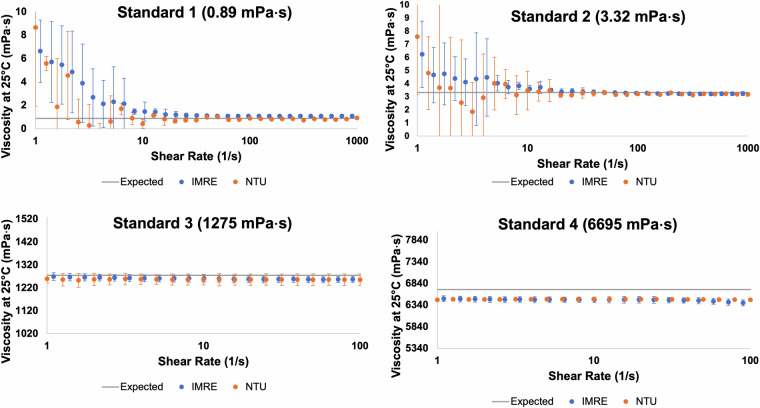
Calibration experiments on both the TA DHR 30 (IMRE) and Anton Paar MCR702e (NTU) rotational rheometers with Newtonian fluid standards.

With respect of rheometry experiments, we optimised the set-up of two rotational rheometers, settling on parallel plates with the specific diameters and operating gap widths stated in the Methods section. This was a non-trivial task, as we had a wide-range of expected viscosities (from water-like samples >1 mPa·s to viscous shampoo formulations ~5,000 mPa·s) to measure with a single rheometry protocol (to maintain consistency with the dataset generation). The operating limits of a rheometer are dictated by its torque sensitivity. For non-viscous samples, one would typically use a Couette cell configuration (“cup-and-bob”) which is more suitable at the lower viscosity range due to the geometry’s extra surface area. However, for the more gel-like samples expected at the upper viscosity range, a “cup-and-bob” is not suitable, and parallel plates are better across the study. We used relatively larger diameter parallel plates to ensure accuracy at the lower viscosity limit. Our calibration runs were with DI water (standard 1) and three general-purpose Newtonian fluid viscosity standards (Paragon Scientific™); results are shown in Fig. 6. The average viscosity over three repeats and error bars corresponding to a 95% confidence interval are shown. We see for the two more viscous standards (1,275 and 6,695 mPa·s) strong agreement on both rheometers with the expected viscosities for the full range of shear-rates, however, the measurements are noisy and only typically settling to an accurate steady-state value after a shear rate of ~10 s^−1^ for the two non-viscous standards (0.89 and 3.32 mPa·s).

At higher shear rates, a greater stress is exerted back on to the plate, and therefore, for the non-viscous standards (which are close to the rheometer’s lower sensitivity limit at low shear rates), their viscosity can be more accurately determined at the higher shear-rates. Overall, if we look at the measured viscosity at a fixed high shear rate, *e.g*., 100 s^−1^, then both rheometers are within 3% of the ground truth across the viscosity range of our prepared liquid formulations.

**Fig. 7:**
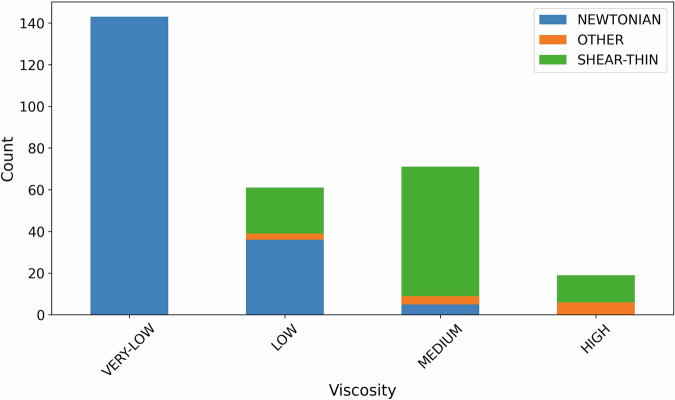
Liquid formulations viscosity measurements grouped by their “zero shear-rate” viscosity into four categories: very low (≤10 mPa·s), low (10 < *η* ≤100 mPa·s), medium (100 < *η* ≤1000 mPa·s), or high (>1000 mPa·s), and their rheology type.

Figure 7 shows viscosity results grouped by their rheology type (Newtonian, shear-thinning, or another type of non-Newtonian fluid). Nearly 50% of the formulations have a water-like low viscosity and these are all classified as Newtonian formulations. We were unable to resolve the viscosity-shear rate profile for these non-viscous samples with sufficient accuracy at low shear rates, as seen in Fig. 6, to be able to make a more detailed deduction of the sample’s rheology. However, it is also known from our domain expertise that it is highly unlikely for such a non-viscous formulation to present a non-Newtonian behaviour, and therefore, this is acceptable. Conversely, Fig. 7 shows that the more viscous the formulation, the greater the proportion of non-Newtonian samples.

In conclusion, our dataset is statistically diverse in its input features (formulation ingredient concentrations), which allows for accurate predictions across the tested design space, and the resulting samples encompass the entire spectrum of property targets explored in our study, including the preferred product profile articulated by our industrial collaborator: a stable, low turbidity, highly viscous, and shear-thinning sample. Notably, this dataset offers sample-specific uncertainty measurements, which are valuable for training more informative surrogate models. Furthermore, these uncertainties are lowest in the regions of greatest interest, low turbidity and high viscosity.

## Usage Notes

**Fig. 8:**
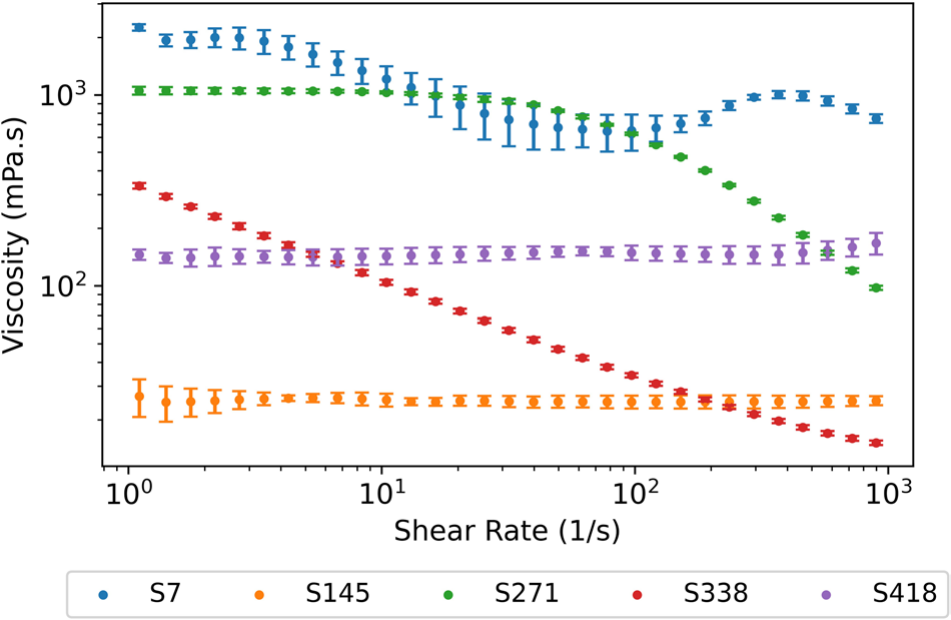
Viscosity – shear rate profiles of selected samples highlighting the rheological behaviours observed from low to high viscosity, and from Newtonian to non-Newtonian fluids.

In this work, we present the formulations dataset as a single JSON file that can be read using all major programming languages (*e.g*., Python, MATLAB, R *etc*.) and a supplementary image dataset, both uploaded publicly at^45^. We provide a code snippet in the SI to read the JSON file into a Pandas dataframe (Python), and further, how to unnest the rheology data so that you can further study the viscosity – shear rate profiles for each sample, as shown for the illustrative examples in Fig. 8. There is detailed information beyond the simple classifications presented as “Viscosity” and “Rheology_Typev”, which are of interest to rheologists and formulators alike.

In summary, we have presented a large open-source dataset of liquid formulations. To generate this dataset, we developed a specific workflow and several underlying methods/experiments. We envisage the primary use case of this work to train property prediction models for liquid formulations (“the forward model”), and later to tackle the inverse design problem. Furthermore, this work is broadly useful to computational materials scientists/chemists, as it is a high-dimensional experimental dataset with multiple conflicting target objectives which can be used to benchmark ML methods for discovery and optimisation.

### Supplementary information

Supplementary Information

## Data Availability

Notebooks and scripts for the automated viscous liquid handling and gravimetric analysis to prepare and determine the composition of the liquid formulations can be found at: https://github.com/sustainable-processes/formulations-prep. The code to run the pHbot has been published separately: https://github.com/sustainable-processes/pHbot^31^. And all the software to develop the computer vision phase stability classifier can be found: https://github.com/sustainable-processes/stability-computer-vision.
