# Supplementary material for: Accelerating Formulation Design via Machine Learning: Generating a High-throughput Shampoo Formulations Dataset
Source: Sci Data. 2024 Jul 3;11:728. doi: 10.1038/s41597-024-03573-w (PMC11222379; doi:10.1038/s41597-024-03573-w)
Supplement: Supplementary file 1 — Supplementary Information [file 41597_2024_3573_MOESM1_ESM.pdf]

Supplementary Information:
Accelerating Formulation Design via Machine Learning: Generating a High-throughput Shampoo Formulations Dataset

Aniket Chitre,[1,2,3] Robert C.M. Querimit,[3,4] Simon D. Rihm,[1,2] Dogancan Karan,[2] Benchuan Zhu,[5] Ke Wang,[5] Long Wang,[5] Kedar Hippalgaonkar[3,6*] and Alexei A. Lapkin[1,2*]

[1] Department of Chemical Engineering and Biotechnology, University of Cambridge, Philippa Fawcett Drive, Cambridge CB3 0AS, United Kingdom

[2] Cambridge Centre for Advanced Research and Education in Singapore, CARES Ltd. 1 CREATE Way, CREATE Tower #05-05, Singapore 138602, Singapore

[3] Institute of Materials Research and Engineering, Agency for Science, Technology and Research (A∗STAR), Singapore 138634, Singapore

[4] School of Chemistry, Chemical Engineering and Biotechnology, Nanyang Technological University, Singapore 637459, Singapore

[5] BASF Advanced Chemicals Co. Ltd., No. 300, Jiang Xin Sha Road, Pudong, Shanghai 200137, China

[6] School of Materials Science and Engineering, Nanyang Technological University, Singapore 639798, Singapore

*Corresponding author email address: kedar@ntu.edu.sg, aal35@cam.ac.uk

Rheology of Formulation Ingredients

We present the rheologies of the formulation ingredients in Figure S1, as measured on the TA Instruments DHR 30 rotational rheometer (with the protocol stated in the Methods section), highlighting the wide range and high viscosity of the ingredients we worked with. This led to notable challenges with viscous liquid handling, which we solved through using our retrofitted Opentrons OT-2 liquid handler and gravimetric analysis protocol, as discussed below.

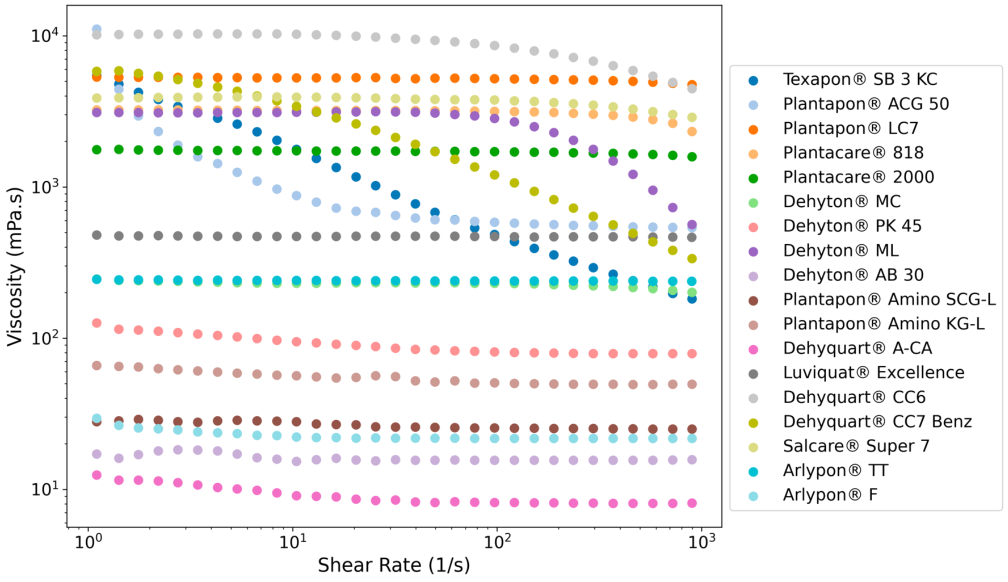

Figure S1. Rheology of the eighteen industrial formulation ingredients used.

Automated Viscous Liquids Handling and Gravimetric Analysis

The first linked GitHub repository in the code availability section (https://github.com/sustainable-processes/formulations-prep) hosts the notebooks and scripts for preparing the liquid formulations. This includes the protocol ("OpentronsProtocol_FormulationSamples.py") to run the Opentrons OT-2 robot, as well as the notebook ("OT-2_BalanceAutomation.ipynb") to log data from the mass balance which we have retrofitted underneath our OT-2. The resulting output from a run, which produces six formulations in one batch, is a CSV file of the mass *vs.* time profile, as shown in Figure 2b. This is taken as input to the "balance_analysis.ipynb" notebook, which calls upon a script ("PipettingMassBalance.py") to deconvolute the step changes in the mass *vs.* time profile, which correspond to ingredient additions, into the formulation compositions. Both the Opentrons Protocol and balance analysis codes read a design of experiments (DoE) CSV file for a particular batch of formulation, which encodes the instructions of the desired ingredient mass fractions for each sample. Due to the challenges of viscous liquid handling, as discussed in the main text, the dispensed ingredient amounts do not match exactly with the desired amounts. It is our task to back-calculate the prepared formulation compositions.

Figure 2b shows the mass *vs.* time profile from an example run in purple. As the mass goes from zero to between approximately forty to fifty grams, this represents the addition of water, which is the base of the shampoo formulations, typically around 65 – 85 w/w% of the total

formulation. Then we have the addition of the surfactants, followed, by the addition of the conditioning polymer, which generally as the most viscous class of ingredient (see Luviquat® Excellence, Dehyquart® CC6, Dehyquart® CC7 Benz and Salcare® Super 7 in Figure S1), requires longer aspiration/dispense times and more blowout cycles (in the OT-2 Protocol), thus leading to protracted step on the mass profile. Finally, the thickener is added last. Note, this order of addition is important, as our industrial partner suggested adding water → + surfactants → + conditioning polymer → + thickener to maximise the probability of phase stability.

After our run, to calculate the formulations compositions we first need to initialise the "balance_analysis" notebook with the appropriate libraries, DoE file (with IDs of the prepared samples) and measure a baseline level of noise detected up by the scale. In order to now verify the individual masses added, the points in time when a specific addition starts, and ends, need to be identified. The general idea to achieve this is that changes in the first and second derivative of the mass profile beyond a multiple of the baseline noise indicate these points. First, a rolling average of the mass profile is calculated which is standard practice to smoothen the data and get more reliable measures of derivatives without outliers.

The initial step is identifying the end of the water addition, which is continuous so it cannot be assigned to a specific sample. The added mass of water is comparatively large so parameters for rolling average and derivative thresholds do not influence the detection. This transfer is taken to be accurate, but we print out the percentage error in dispensing water, just to check. This is almost always within an acceptable 1% deviation. Next, we need to identify the ingredient additions that are done in single pipetting steps that need to be separated. This poses a challenge not only because the frequencies of additions differ but also because some of the ingredients are highly viscous so that an additional drop will be registered a while after the initial addition. As mentioned in the main text, different liquid handling parameters were used for the surfactants, conditioning polymers, and thickeners. Therefore, our signal processing algorithm ("PipettingMassBalance.py") defines a method with three key parameters: (i) `avg_window` = the period to compute a rolling average of the mass profile over; (ii) `bl_mult` = a multiple of the baseline noise, which is the threshold the first and second derivative of the mass profile must exceed to be counted as an ingredient addition peak; (iii) `mergeSens` = a merge sensitivity parameter to understand when to consider a peak as distinct, or for example, a trailing droplet of the previous peak.

Figure S2 shows a zoomed-in part of the mass profile, resolving the ingredient additions for the conditioning polymers, as an illustrative example of the method. We see the smoothed signal computed from a rolling average of the raw mass profile and our method relies on looking at where the first and second derivatives (in green and blue, respectively) cross the threshold, defined as discussed, as some multiple of the baseline noise, dependent on the type of ingredient addition being analysed (surfactant/conditioning polymer/thickener).

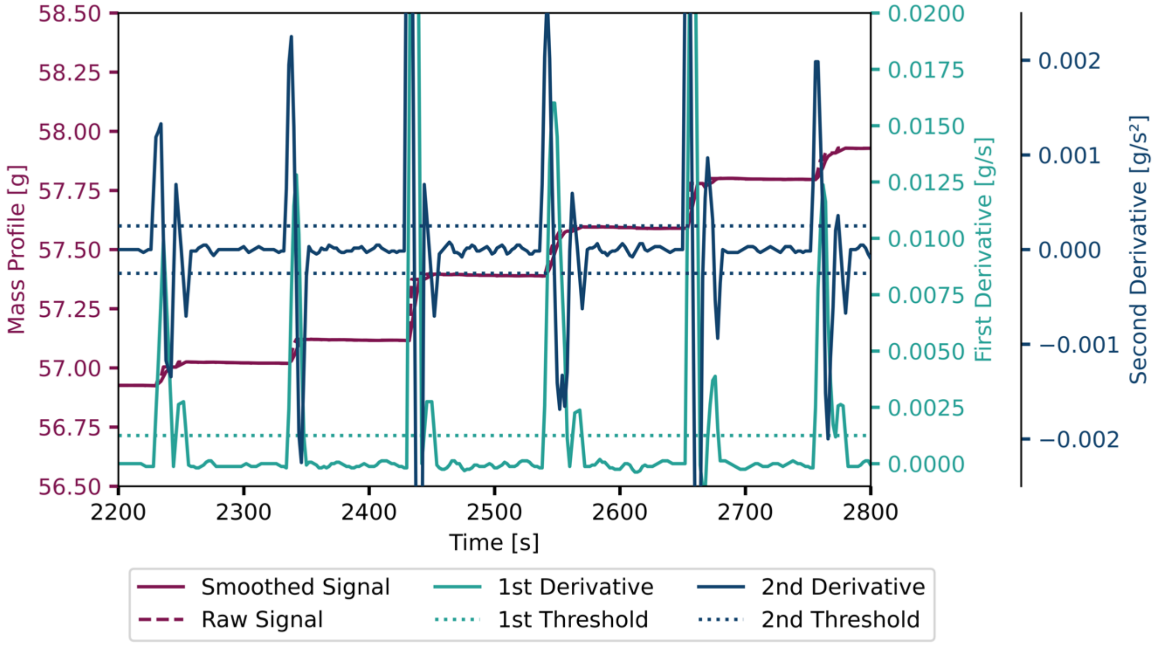

Figure S2. Gravimetric analysis method to resolve the ingredient additions by identifying the start and end times of a dispense through an inspection of the first and second derivatives of the mass profile with respect to time, used to back-calculate the formulation compositions.

Finally, we note, the max capacity of a single transfer was 1 mL, however, our max requested dispenses for the surfactants could exceed this amount, in which case our Opentrons Protocol handled splitting the transfer up into steps, and our mass analysis method had to take this into account too. Overall, we would end up with a list of dispense steps with their measured amounts and could reindex this into a Pandas dataframe (using the DoE steps) to present the formulation compositions in a format which could be directly appended to the overall formulation dataset.

Imaging Formulation's Phase Stability

We show the imaging station which we set up to capture the formulation images in Figure S3. The "webcam_image.py" file on: https://github.com/sustainable-processes/stability-computer-vision was used to control the webcam, which was operated at a fixed focus and in a lightbox.

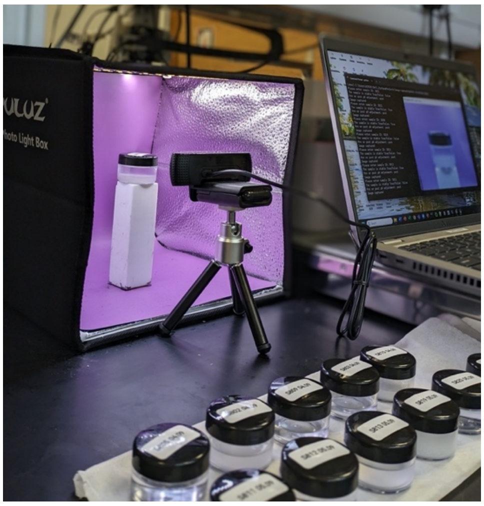

Figure S3. Formulations imaging station – Logitech C922 webcam controlled by open-cv imaging samples at a fixed position within a lightbox.

Proxy Turbidity Measurement

We show in Figure S4 the original calibration points from the turbidity standards, and intermediates of their mixtures, purchased from Sigma Aldrich. The x-error bars represent the 95% confidence interval in the measured UV-absorbance at 420 nm with the MTP stated in the Methods section. We have fit a GP regressor as the calibration curve through the points.

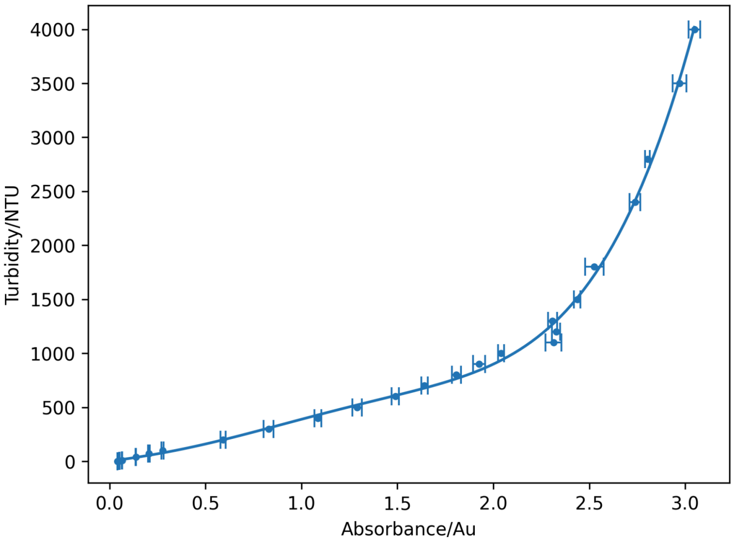

Figure S4. Turbidity – absorbance calibration curve which is fit with a GP regressor model.

Budgeting for an Automated Formulations Lab

Table S1 shows the breakdown of costs to reproduce the workflow presented under the Methods section. As we intended to best utilise existing equipment from our laboratory/institute and then complement that with lower-cost robotics as necessary, we include a "Status" column in Table S1 to show where we purchased new equipment specifically for this project – the costs of these items total approximately 15,000 USD. We developed a high-throughput proxy for measuring turbidity using a pre-existing plate reader and performed offline viscosity measurements on an available rotational rheometer, which was the bottleneck for the workflow and would be the cost-limiting factor if one had to be purchased. For such high capital cost equipment, we have estimated in Table S1 either purchasing time as an external user or buying a simpler viscometer, which would, however, have more limited functionality. The total costs are thus estimated closer to 40,000 USD.

Table S1. Estimated costs to develop a high-throughput liquid formulations workflow.

Item	Estimated Cost (USD)	Status	Notes
Liquid Handling Robot (Opentrons OT-2)	10,000	Pre-existing	Our lab procured its first OT-2 for ~ 5k USD in 2019.
Mettler Toledo Precision Balance	5,000	Purchased	Integrated with cheap prototyping (3D-printing) into our OT-2 robot.
IKA RT15 Multi-position Hotplate Stirrer	5,000	Purchased	The heating element was not required here, but useful to have.
Webcam and Lightbox	100	Purchased	We put together an imaging station for stability analysis.
pHbot	5,000	Built in-house with purchased /existing parts	A full bill of materials is provided in the SI of our pHbot article[31] – the most significant cost is for high precision syringe pumps

			which we re-utilised from another project.
UV-Vis Microplate Reader	5,000	Pre-existing	We had a high-end Tecan Infinite M200 plate reader, but a simpler model will do.
Rotational rheometer*	10,000	Pre-existing	*We used a rheometer available within the institute at a reduced internal rate. We have roughly costed externally purchasing time on a rheometer or buying a viscometer.
Consumables (Pipette Tips, Sample Jars *etc.*)	500	Purchased	Purchased cosmetic jars in bulk from AliExpress.
Chemicals (ingredients, titrants, standards *etc.*)	500	Purchased	Formulation ingredients were sent as samples from our industry partner.
Misc. (*e.g.*, Pos-D pipette)	400	Purchased	
**Total**	**~ 42k USD**		

Surfactants Alkyl Chain Length Characterisation

Chain length characterisation was performed in UPLC-MS (Agilent 1260 Infinity II LC-MS) equipped with Agilent XDB-C18 3.5 µm, 3 x 100 mm column. Samples were prepared by dissolving 3 - 5 µL surfactant ingredient in 1 mL mobile phase mixture (95:5 v/v water: acetonitrile with 0.1 % trifluoroacetic acid). Injection volume from the autosampler was 1 µL. Molecular weight distribution was resolved by using the following quaternary pump protocol: The initial mobile phase mixture was 5:95 binary mixture of acetonitrile and water (with 0.1 % trifluoroacetic acid) flowing at 0.6 mL/min. After the sample injection, the flow rate ratio of the acetonitrile/water mixture steadily changed to 95:5 over 2.5 minutes. Next, the flow rate ratio was returned to 5:95 over a duration of 5 minutes. Finally, the flow rate ratio was held constant for 0.5 minutes after which the analysis was completed. Total analysis time for one

sample was 8 minutes. Following MS parameters were used in the analysis: drying gas flow rate = 12 ml/min, nebuliser pressure = 35 psig, drying gas temperature = 350 °C, capillary voltage = 3000 V, Mass range 100 – 1000 g/mol. Both positive and negative channels were used to capture all the ions. Molecular weight distribution of surfactants (Figure S5) was estimated in a semi-quantitative manner by the area normalization of UPLC-MS chromatograms. However, the molecular weight distribution of Plantapon® LC 7 and Dehyquart® A-CA could not be resolved by this method due to the complete fragmentation of the structure in MS. Therefore, their structure was assumed to be 100 % of the structure shared by our industrial partner.

In Figure S5 C_0 refers to the original ingredient structure provided to us by our industrial partner and included in the slides. The alkyl chain of the surfactants could be longer or shorter by *x* CH_2 units, where C_neg1 means one CH_2 unit fewer, or C_pos1 one CH_2 unit more *etc.* Figure S5 shows that except for a couple of ingredients, the major product is the one shared by our industrial partner, however, there is a significant distribution, which should be accounted for in future molecular modelling studies, as the alkyl chain length is one of the most important factors governing a particular surfactant's behaviour.

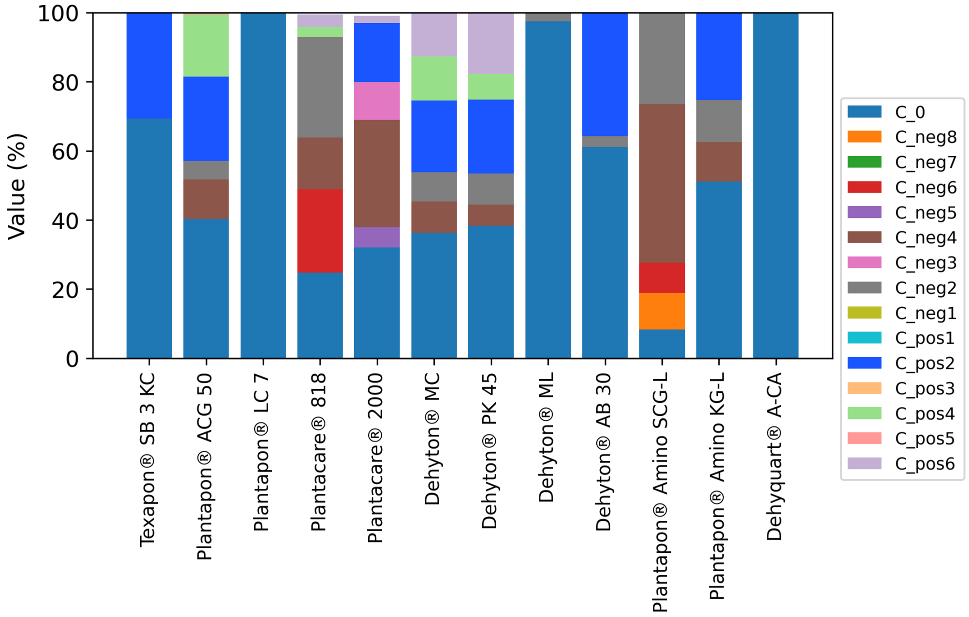

Figure S5. Surfactant ingredients alkyl chain distribution.

Distribution and Diversity of Ingredient Concentrations

The input features for the dataset are the ingredient concentrations. Upon agreement with our industrial partner, we aimed to add approximately between 8 – 13 w/w% surfactant, 1 – 3 w/w% polymer, and 1 – 5 w/w% thickener in each formulation, with the rest being water. In Figure S6 we show the distribution of surfactant concentrations, which for the particularly viscous ingredients (*e.g.,* Texapon® SB 3 KC or Plantapon® LC 7) could be significantly underdispensed. By contrast, if we select a non-viscous ingredient (*e.g.,* Dehyquart® A-CA, see Figure S1) our dispenses are within the expected bounds. Either way, this is not an issue for our data generation campaign, because as long as we use a diverse range of ingredients and concentrations, which we can see from the well-distributed histograms in Figure S6, we do, then we will generate a dataset that meets our aims and objectives.

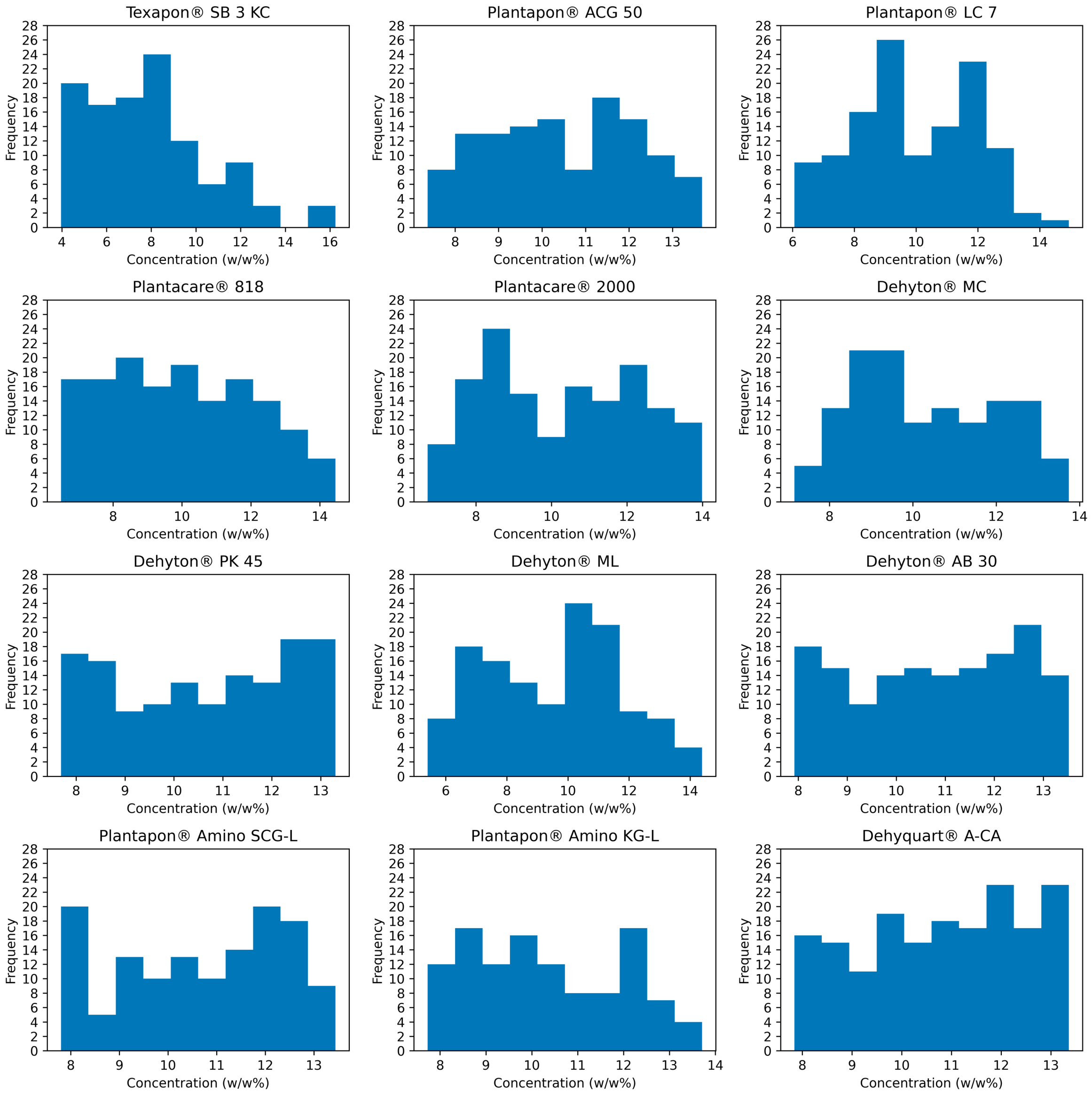

Figure S6. Distribution of surfactant concentrations across the formulations dataset.

We present Figure 3 and discuss in the main text that the ingredient additions are not correlated with each other and therefore, the design space has been well explored. We further support this with Figure S7 where this pair plot explores the correlation between each pair of ingredients in this 18 x 18 grid, plus the off-diagonal shows the 1-d concentration distribution for each ingredient. We can see no discernible correlation between the points in any individual grid, and the points typically fall within a square or rectangular shape, dictated by the concentration bounds mentioned above.

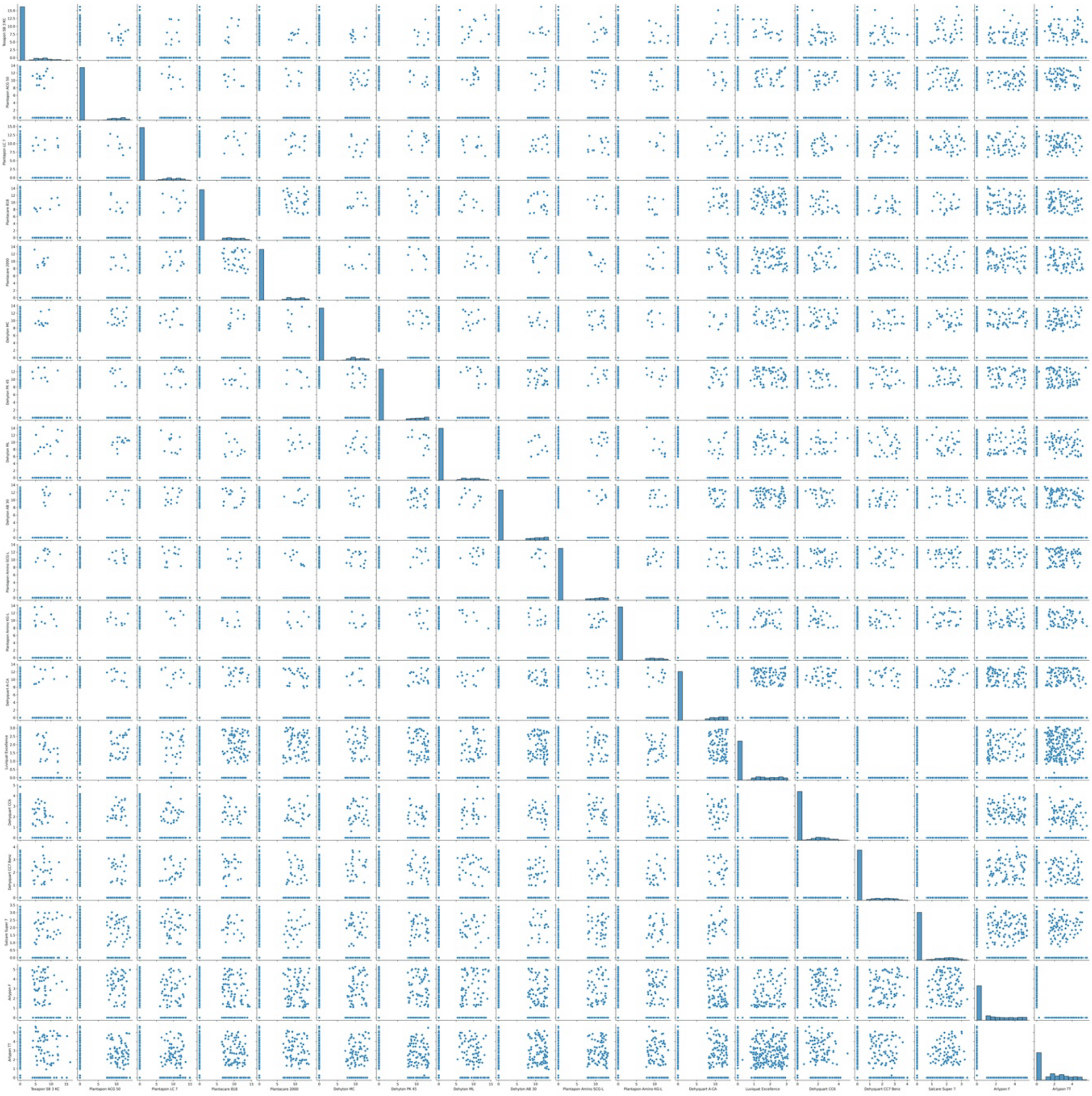

Figure S7. Pair plot for the formulation ingredients across the dataset.

Working with the JSON dataset in Python Panadas

```
df = pd.read_json("LiquidFormulationsDataset_2023.json")
df.set_index("ID", inplace=True)

df_stable = df[df['Stability_Test'] == True] # filter out stable formulations

# Unpack the Rheology_Data column
df_rheology =
pd.json_normalize(df_stable["Rheology_Data"]).set_index(df_stable.index)
df_rheology.rename(columns={0: 'shear_rate', 1:'avg_viscosity', 2: 'std_dev'},
inplace=True)

# Clean up the DataFrame
df_rheology['shear_rate'] = df_rheology['shear_rate'].apply(lambda x:
x['shear_rate'])
df_rheology['avg_viscosity'] = df_rheology['avg_viscosity'].apply(lambda x:
x['avg_viscosity'])
df_rheology['std_dev'] = df_rheology['std_dev'].apply(lambda x: x['std_dev'])
```
